# Biogeography of Cyanobacterial *isiA* Genes and Their Link to Iron Availability in the Ocean

**DOI:** 10.3389/fmicb.2019.00650

**Published:** 2019-04-04

**Authors:** Qian Li, Jef Huisman, Thomas S. Bibby, Nianzhi Jiao

**Affiliations:** ^1^State Key Laboratory of Marine Environmental Sciences, Institute of Marine Microbes and Ecosphere, College of Ocean and Earth Sciences, Xiamen University, Xiamen, China; ^2^Department of Freshwater and Marine Ecology, Institute for Biodiversity and Ecosystem Dynamics, University of Amsterdam, Amsterdam, Netherlands; ^3^Center for Microbial Oceanography: Research and Education, Department of Oceanography, University of Hawai’i at Mānoa, Honolulu, HI, United States; ^4^School of Ocean and Earth Science, National Oceanography Centre Southampton, Faculty of Natural and Environmental Sciences, University of Southampton, Southampton, United Kingdom

**Keywords:** cyanobacteria, *Synechococcus*, iron limitation, antenna protein, *isiA*, chlorophyll

## Abstract

The cyanobacterial iron-stress-inducible *isiA* gene encodes a chlorophyll-binding protein that provides flexibility in photosynthetic strategy enabling cells to acclimate to low iron availability. Here, we report on the diversity and abundance of *isiA* genes from 14 oceanic stations encompassing large natural gradients in iron availability. *Synechococcus* CRD1 and CRD2-like *isiA* genes were ubiquitously identified from tropical and subtropical waters of the Pacific, Atlantic, and Indian Oceans. The relative abundance of *isiA*-containing *Synechococcus* cells ranged from less than 10% of the total *Synechococcus* population in regions where iron is replete such as the North Atlantic subtropical gyre, to over 80% in low-iron but high-nitrate regions of the eastern equatorial Pacific. Interestingly, *Synechococcus* populations in regions with both low iron and low nitrate concentrations such as the subtropical gyres in the North Pacific and South Atlantic had a low relative abundance of the *isiA* gene. Indeed, fitting our data into a multiple regression model showed that ∼80% of the variation in *isiA* relative abundances can be explained by nitrate and iron concentrations, whereas no other environmental variables (temperature, salinity, Chl *a*) had a significant effect. Hence, *isiA* has a predictable biogeographical distribution, consistent with the perceived biological role of IsiA as an adaptation to low-iron conditions. Understanding such photosynthetic strategies is critical to our ability to accurately estimate primary production and map nutrient limitation on global scales.

## Introduction

Picocyanobacteria belonging to the *Synechococcus* genus are among the most abundant photosynthetic organisms in open ocean ecosystems (Partensky et al., 1999; Scanlan and West, 2002) and are among the key contributors of primary production (Liu et al., 1998; Flombaum et al., 2013) and carbon cycling (Guidi et al., 2016). Their biogeography is associated with different habitats driven by several major abiotic factors including light (Grébert et al., 2018), temperature (Zwirglmaier et al., 2008), macronutrients and iron (Sohm et al., 2015; Farrant et al., 2016). The versatility of light-harvesting systems in the *Synechococcus* genus has substantially contributed to their ability to adapt to different underwater light environments (Stomp et al., 2004; Six et al., 2007) and exploit different ecological niches (Ting et al., 2002; Scanlan et al., 2009; Grébert et al., 2018). Their ability to change photosynthetic strategy in response to variation in iron availability (Bibby et al., 2001) is of particular interest as iron limits marine primary production in large oceanographic regions (Martin and Fitzwater, 1988; Behrenfeld et al., 1996; Boyd et al., 2000).

The chlorophyll-binding IsiA protein complex has been demonstrated to play a central role in the light-harvesting strategy of many cyanobacteria (Boekema et al., 2001; Yeremenko et al., 2004; Snow et al., 2015). Expressed by the iron-stress-inducible *isiA* gene of cyanobacteria (Bibby et al., 2001; Geiss et al., 2001a), the IsiA proteins form six-transmembrane helices, bind chlorophyll and form photosynthetic ‘supercomplexes’ with the reaction centers of photosystem I (PSI) (Bibby et al., 2001). Most knowledge of the IsiA protein has been acquired by the study of model cyanobacteria like the freshwater species *Synechococcus* PCC 7942 (Ghassemian and Straus, 1996; Boekema et al., 2001) and *Synechocystis* PCC 6803 (Yeremenko et al., 2004; Havaux et al., 2005). However, the gene has also been identified and characterized widely in marine cyanobacterial species (including *Synechococcus, Prochlorococcus*, and other marine *Cyanobacterium* spp.), through experiments conducted under iron limitation (Leonhardt and Straus, 1992; Bibby et al., 2003; Hewson et al., 2009; Richier et al., 2012), genomic (Palenik et al., 2006; Dufresne et al., 2008; Rivers et al., 2009) and metagenomic analyses (Bibby et al., 2009). Although *isiA* may be expressed under other growth conditions, including high salt (Vinnemeier et al., 1998), high heat (Vinnemeier et al., 1998; Kojima et al., 2006) and high light (Havaux et al., 2005), acclimation to iron stress appears to be the primary functional role for this chlorophyll binding complex (Ghassemian and Straus, 1996; Bibby et al., 2001; Ryan-Keogh et al., 2012).

As a single copy gene, not all oceanic strains of *Synechococcus* possess an *isiA* gene (Rivers et al., 2009; Scanlan et al., 2009). The non-*isiA*-harboring *Synechococcus* str. WH8102 and *isiA*-harboring str. WH8020 were isolated from the south Sargasso Sea (high dust iron input) and coastal New England shelf (highly seasonal iron dynamics), respectively, which suggests the presence of *isiA* in a genome might be an adaptation response to consistent low or variable iron conditions. Indeed, comparative genomics has identified *Synechococcus* str. WH8020 tend to have more iron-responsive genes in general compared to str. WH8102 (Mackey et al., 2015).

The IsiA-PSI supercomplex increases the functional absorption cross-section of PSI under iron stress conditions (Bibby et al., 2001; Boekema et al., 2001; Ryan-Keogh et al., 2012). However, not all of the chlorophyll associated with IsiA is photosynthetically active and overexpression of IsiA uncoupled to photosynthetic reaction centers is commonly observed when iron is depleted and macronutrients are replete (Schrader et al., 2011; Ryan-Keogh et al., 2012). There is evidence that both prokaryotic and eukaryotic phytoplankton species employ similar photosynthetic strategies that result in accumulation of energetically uncoupled chlorophyll under iron stress, which can comprise as much as 40% of total chlorophyll (Macey et al., 2014). These disconnected pools of chlorophyll elevate fluorescence yield, making it possible to map regions of iron limitation remotely (Behrenfeld et al., 2006, 2009; Lin et al., 2016). Knowledge of the biogeographical distribution of these chlorophyll-binding antenna systems, especially under high-nutrient low-chlorophyll (HNLC) conditions, is central to our ability to accurately interpret satellite chlorophyll data, to estimate ocean primary production, and to identify oceanic regions with potential iron limitation (Behrenfeld et al., 2006, 2009; Behrenfeld and Milligan, 2013; Lin et al., 2016).

Targeted studies on the diversity and biogeographical distribution of *isiA* genes in the global ocean, and their association with iron limitation, are scarce. In this study, we designed *isiA* gene primers specific for marine *Synechococcus* spp., and investigated the biogeographical distribution, diversity and abundance of *isiA* genes from 14 oceanic stations that differ widely in iron availability using clone libraries and qPCR. In particular, we aimed to derive a relationship between the abundance of the *isiA* gene and iron availability, which would enable the use of *isiA* as a genetic indicator of iron limitation in marine cyanobacteria.

## Materials and Methods

### Sample Collection

**FIGURE 1:**
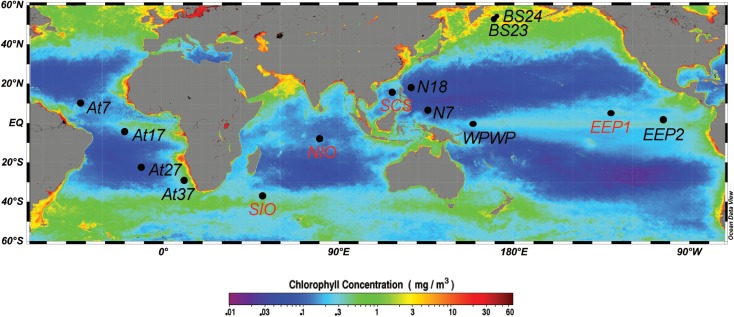
Map showing the 14 sampling stations, including eight surface stations (black labels: BS23, BS24, N18, N7, At7, At17, At27, At37) and four vertically resolved stations (red labels: SCS, EEP1, SIO, NIO). Sampling sites were mapped using Ocean Data View (Schlitzer, 2012), with the base map of annually averaged (2008) surface chlorophyll *a* concentration extracted from the NASA website (http://oceancolor.gsfc.nasa.gov/).

Water samples were collected between 2008 and 2011, onboard the R/V Da Yang I, R/V Ke Xue I, and R/V Dong Fang Hong II (Table 1). In total, we sampled 14 stations (Figure 1), including one station in the South China Sea (SCS), three stations in the equatorial Pacific Ocean (two in the typical HNLC eastern equatorial Pacific Ocean, EEP1 and EEP2; and one in the western Pacific warm pool, WPWP), two stations in the western part of the North Pacific subtropical gyre (N18 and N7), two stations in the Indian Ocean (NIO and SIO), four in the Atlantic Ocean (At7, At17, At27, and At37), and two in the Bering Sea (BS23 and BS24). At all stations, samples were taken from just below the water surface (5 m depth) using a rosette sampler with 20-L Niskin bottles. In addition, at four stations (SCS, EEP1, SIO, and NIO) we investigated a depth profile with samples from 5, 50, 100, and 200 m depth. From each sample, 2-L of water was filtered through 0.22-μm pore size 47-mm diameter polycarbonate filters (Millipore, Bedford, MA, United States), and immediately frozen and stored at -80°C until DNA extraction. An extra 2-mL of seawater was sampled in triplicate, fixed with glutaraldehyde (final concentration 0.1%, Marie et al., 1997), quick-frozen in liquid nitrogen and stored in a freezer at -20°C for later flow cytometry analysis.

**Table 1:** Sampling information and relevant environmental parameters in the surface water of each station.

Region	Station	Depth	Lat.	Long.	Date	Temp (°C)	Sal. (psu)	Chl *a* (μg/L)	DFe^a^ (nM)	NO_3_^b^ (μM)	[NO_3_]:[DFe]^c^ (μM:nM)
Eastern Pacific	EEP1	5	6.0N	129.9W	2011/3/11	27.4	34.5	0.2	0.12	4.1	33.5


		50				27.3	34.6	0.5	0.15	5.3	35.6
		100				24.4	34.8	0.6	0.22	6.4	29.8
		200				17.8	34.4	0.1	0.63	12.6	20.0
	EEP2	5	1.8N	102.3W	2011/5/10	25.8	33.5	0.3	0.12	5.4	43.9
Indian Ocean	SIO	5	37.7S	50.8E	2010/18/2	21.2	35.0	0.4	0.30	3.8	12.7


		50				20.9	35.1	0.8	0.39	6.8	17.5
		100				17.8	35.0	0.3	0.46	7.1	15.7
		200				11.2	34.9	0.1	0.68	10.4	15.2
	NIO	5	8.0S	80.0E	2010/24/12	26.2	35.0	0.2	0.63	3.8	6.1
		50				25.1	34.9	0.3	0.91	7.5	8.2
		100				21.1	34.5	0.3	0.68	13.6	20.0
		200				18.0	34.4	0.2	0.80	13.8	17.2
Atlantic Ocean	At7	5	10.6N	42.3W	2008/11	27.1	35.4	0.1	1.54	0.1	0.1


	At17	5	3.8S	21.3W	2008/2/11	26.3	36.0	0.2	0.88	3.5	3.9
	At27	5	22.1S	9.6W	2008/11	20.2	35.6	0.1	0.37	0.0	0.1
	At37	5	27.6S	12.9E	2008/11	18.5	35.0	0.5	0.40	6.1	15.3
SCS and Western Pacific	SCS	5	16.5N	117E	2011/23/9	29.4	33.8	0.1	0.54	0.4	0.7


		50				27.9	34.1	0.3	0.58	1.4	2.4
		100				23.2	34.4	0.03	0.71	2.2	3.0
		200				17.3	33.9	0.01	0.73	4.0	5.5
	N18	5	18N	126E	2010/10/7	28.9	34.1	0.1	0.25	0.0	0.2
	N7	5	6.5N	135E	2010/12/7	29.1	33.5	0.2	0.18	0.0	0.2
	WPWP	5	0	160E	2008/30/12	29.6	34.8	0.3	0.14	1.9	13.9
Bering Sea	BS23	5	53.3N	169.9E	2008/24/7	8.0	35.2	0.3	0.44	5.3	11.9
	BS24	5	54.2N	170.5E	2008/24/7	8.5	34.5	0.3	0.50	4.4	8.8


*^*a,b*^Averaged value of [DFe] and [NO_*3*_] collected from different model simulations and both model simulation and *in situ* measurements, respectively. See details in Supplementary Table S1. ^*c*^Average [NO_*3*_]:[DFe] ratios obtained by dividing average [NO_3_] by the average [DFe].*

### Environmental Parameter Measurements

Profiles of temperature, salinity, and total chlorophyll *a* at each station were measured with a SeaBird CTD (SBE 9/11 plus, SeaBird Inc., United States) equipped with auxiliary sensors for chlorophyll *a* fluorescence. NO_x_ (nitrate+nitrite) concentrations were measured after reduction of nitrate to nitrite with a Technicon AA3 Auto Analyzer (Braun-Lube, GmbH) using standard protocols (Grasshoff et al., 1999) (Table 1). Nitrite concentrations were measured similarly but without the reduction step and subtracted from NO_x_ to calculate the nitrate concentration [NO_3_]. The detection limits for NO_x_ were 0.03 μM.

### Defining Iron Environment

Dissolved iron (DFe) concentrations were retrieved from two different models on different time scales (Table 1 and Supplementary Table S1). (1) NASA’s Ocean Biogeochemical Model (NOBM^1^) according to Gregg et al. (2003), from which we retrieved daily (at the sampling day, based on daily simulation), monthly (at the sampling month, based on monthly simulation) and annually averaged (at the sampling year, based on monthly simulation) DFe concentrations at a special resolution of 1.25 × 0.67 degree (longitude-latitude). (2) The Ecco2-Darwin model from which we retrieved the monthly (at the sampling month, based on monthly simulation) and annually averaged (at the sampling year, based on monthly simulation) DFe at 1 × 1 degree (longitude-latitude) (data file available to download^2^) (Behrenfeld et al., 2009; personal communication with Oliver Jahn and Stephanie Dutkiewicz). DFe concentrations at the four stations for which we collected depth profiles were only retrieved from the Ecco2-Darwin model (at both monthly and annually time scale). A caveat of this approach is that the simulated DFe concentrations may not represent the exact DFe concentration *in situ*, and thus may introduce additional variance in the correlation between *isiA* and iron. However, using the mean of two independent models and different time scales could help mitigate some of this variation, and possibly retrieve the closest-to-real iron and nitrogen conditions the ambient *Synechococcus* had been experiencing. To assess the reliability of this modeling approach, we also retrieved NO_3_ concentrations from the models, which matched well with the NO_3_ concentrations measured in our study (Supplementary Table S1). Therefore, the averaged DFe and NO_3_ concentrations based on two models at two or three simulation time scales and the NO_3_ *in situ* measurements were used for our statistical analysis.

The molar ratio of [NO_3_]:[DFe] is widely used in biogeochemistry models (Fung et al., 2000; Tagliabue et al., 2016; Browning et al., 2017) to indicate iron and/or nitrate limitation. It is thus applied for this study to characterize relative iron availability as it also describes conditions where nitrate and iron are co-limiting (Moore et al., 2013; Browning et al., 2017). Available observational data from earlier studies near our sampling stations show that this ratio provides a good indication of nutrient limitation patterns in the oceans (Supplementary Table S2). More specifically, [NO_3_]:[DFe] ratios <1 μM/nM indicate that nitrate is the primary limiting nutrient, whereas ratios >10 (Browning et al., 2017) or >5 μM/nM (Biller and Bruland, 2014) suggest iron is primarily limiting.

### *Synechococcus* Abundance

Abundances of *Synechococcus* were determined using 2-mL seawater aliquots on an Epics Altra II flow cytometer (Beckman Coulter, Brea, CA, United States) with a 306C-5 argon laser (Coherent, Santa Clara, CA, United States), according to the method of Jiao et al. (2002). They were identified and distinguished from other autotrophs based on their positions in plots of side scatter versus red fluorescence and orange fluorescence versus red fluorescence. Event rates were set to 50–200 events/second (0.1–1 mL h^-1^) in order to enhance the particle capturing sensitivity. Fluorescent microspheres of 1 μm diameter (Polysciences Inc., Warrington, PA, United States) were added to all samples as an internal standard to calibrate flow rate and cell size. All samples were run in triplicate. The data were analyzed with EXPOTM32 MultiCOMP software (Beckman Coulter, United States).

### DNA Extraction, PCR Amplification, Cloning, and Sequencing

The genomic DNA of the bacterial community was extracted using a commercial fast DNA kit (Qiagen, United States). Software package Primer 5.0 and Geneious R10 (Biomatters Ltd., United States) was used to generate new *isiA* primers targeting *Synechococcus* spp. based on the conserved protein sequences. Detailed information on the primer design is given in the “Results” section, Supplementary Figures S1, S2 and a collection of all *isiA* and *pcb* genes used as references is summarized in Supplementary Table S3.

The new degenerate primer pair *isiA*33F (5′-ACY TAT GAC TGG TGG GC-3′) and *isiA*654R (5′-CCD CCC ATV ACR TCY TC-3′) (with variable nucleotides Y = C/T, D = A/G/T, V = A/G/C, and R = A/G) was then tested for specificity and cross-reactivity with marine *Synechococcus* str. CC9605 (as a positive control), str. WH8102 (as a negative control) and field samples from station SIO. A predicted ∼625 bp fragment was obtained by PCR performed with Ex Taq PCR Mix (TaKaRa, Dalian, China). The PCR program was run in a T3 thermocycler (Biometra, Germany) and consisted of an initial 94°C denaturation step for 5 min, 35 cycles of denaturation at 94°C for 45 s, annealing at 52°C for 45 s and extension at 72°C for 45 s. Amplification products were gel-purified, ligated into pMD18-T vectors (TaKaRa, Dalian, China), and transferred to *Escherichia coli* DH5*α*-competent cells (TaKaRa, Dalian, China). Positive clones were randomly chosen and screened with vector M13-F/M-13R using an ABI model 3730 automated DNA sequence analyzer with BigDye terminator chemistry (Applied Bio Systems, Perkin-Elmer).

### Phylogenetic and Biogeographical Analysis

DNA sequences were analyzed, aligned and translated into protein sequences (∼200 amino acids) using *MEGA* version 6 (Tamura et al., 2013) and Genieous R10 (Biomatters Ltd., United States). Multiple alignments of MAFFT v7.388 E-INS-i (Katoh and Standley, 2013) with BLOSUM62 scoring matrix was used for the sequence alignments. DNA and protein sequences were afterward blasted against the NCBI gene bank using the BLASTN and BLASTP tools to aid the selection of the closest reference sequences. Operational taxonomic units (OTUs) of sequences sharing identities of at least 90% for DNA and 97% for protein were identified and their Shannon diversity index was calculated using the DOTUR program (Schloss and Handelsman, 2005). These thresholds were tested to be most suitable for grouping closely related *isiA*/IsiA phylotypes with different references.

Maximum likelihood phylogenetic trees using 97% identity IsiA protein sequences were then constructed and compared both in *MEGA* using the Gamma LG model and RAxML^3^. Bootstrap analysis was used to estimate the reliability of phylogenetic reconstructions under 100 replicates. The final IsiA protein sequence phylogenetic tree was edited with iTOL^4^ (Letunic and Bork, 2016).

### Quantitative PCR Amplification

The abundance of *Synechococcus isiA* genes was determined using the quantitative PCR (qPCR) method and running with the CFX 96^TM^ real-time system (Bio-Rad, Singapore). The specific primer pair for amplifying *isiA* genes (*isiA*33F, 5′-ACY TAT GAC TGG TGG GC-3′ and *isiA*348R, 5′-CCG DAG DGT RTG CCA GAT-3′; target *isiA* genes between length 33 bp and 348 bp) was designed using the same method as for the PCR primers. These qPCR primers were then checked for specificity and cross-reactivity with *Synechococcus* str. CC9605 (as a positive control) and str. WH8102 (as a negative control). Standard curves were constructed using a mixture of equal amounts of the plasmid DNA of the three most dominant *isiA* phylotype clones (374-WPWP-5m, 440-BS23-5m, and 367-SIO-50m). Using ten-fold increments, the standard concentrations were adjusted from 10^7^ to 10^1^ gene copies μL^-1^ of *isiA*. The reaction mixture (25 μL) contained 12.5 μL SYBR Premix Ex Taq II (TakaRa, Dalian, China), 0.5 μM of each primer and 1 μL template DNA, and qPCR thermal cycling conditions were the same as described for the PCR amplification. The amplification efficiencies of qPCR were always between 80 and 95% with *R*^2^ values > 0.99, and the sensitivity ranged from 10^2^ to 10^8^ *isiA* gene copies L^-1^ in the environmental samples (Supplementary Table S6). The specificity of the qPCR reactions was confirmed by melting curve analysis, agarose gel electrophoresis and sequencing analysis.

### Nucleotide Sequence Accession Numbers

The environmental *isiA* sequences obtained in this study have been deposited in the NCBI GenBank database under accession numbers MF772983–MF773424.

### Statistical Analysis

Redundancy analysis (RDA) was applied to investigate correlations between *isiA* phylotypes and the measured environmental parameters. RDA was performed using forward selection with the Ordistep function in the R package *vegan* (Oksanen et al., 2015) using R version 3.3.3 (R Development Core Team, 2017). Significance was based on a permutation test with 999 permutations to select only those environmental parameters that contributed significantly (*P*_adjusted_ < 0.05) to the RDA model (Zuur et al., 2009).

Multiple regression was used to investigate the relationship between the relative abundance of *isiA*-containing *Synechococcus* (*isiA*:cell, expressed as number of *isiA* gene copies L^-1^ divided by the number of *Synechococcus* cells L^-1^) and the measured environmental parameters. From a biological perspective, we are particularly interested in the hypothesis that iron limitation favors a high relative abundance of *isiA*-containing *Synechococcus* cells. That is, we would like to know whether the *isiA*:cell ratio is negatively correlated with iron availability, but positively correlated with other potentially limiting nutrients. The number of *isiA* gene copies L^-1^ and the number of *Synechococcus* cells L^-1^ were both approximately log-normally distributed, whereas [DFe] and [NO_3_] were normally distributed. We therefore investigated the following multiple regression model:

$$\mathrm{Log}(\mathrm{isiA}:\mathrm{cell})\;=\;a_{1}[\mathrm{DFe}]\;+\;a_{2}[{\mathrm{NO}}_{3}]\;+\;...\;+\;b$$

where we used log base 10. We note that, from a statistical perspective, the use of ratios of two variables can be problematic in regression analysis, because it can lead to spurious correlations (e.g., Kronmal, 1993). However, log(*x*:*y*) is equivalent to log(*x*) - log(*y*), and therefore the above regression model can also be written in a slightly more elaborate form as:

$$\mathrm{Log}(\mathrm{isiA})\;=\;a_{0}\mathrm{Log}[\mathrm{cell}]\;+\;a_{1}\mathrm{[DFe]}\;+\;a_{2}[{\mathrm{NO}}_{3}]\;+\;...\;+\;b.$$

Regression analysis showed that the value *a*_0_ was very close to 1, which supports the use of log(*isiA*:cell) as the response variable in the regression model. A normal quantile–quantile (Q–Q) plot of the standardized residuals was used to assess the goodness-of-fit of the regression model.

## Results

### Geographic Regions and Oceanographic Characteristics

*IsiA* gene diversity and abundance was retrieved from several oceanic environments, including samples from the equatorial Pacific Ocean (EEP1, EEP2, WPWP), equatorial Atlantic Ocean (At17), Northern Indian Ocean (NIO), Southern Indian Ocean (SIO), SCS, and Bering Sea (BS23, BS24) (Table 1 and Figure 1). In addition to surface samples, stations EEP1, SCS, SIO and NIO were sampled over depth profiles to 200 m. Abundance of *isiA* was also investigated in the western part of the North Pacific subtropical gyre (N7, N18), the North Atlantic subtropical gyre (At7) and southern Atlantic (At27, At37), although we did not investigate *isiA* gene diversity at these stations. The stations encompass a wide range of dissolved iron (DFe) concentrations, from <0.2 nM to >1.5 nM (Table 1). The [NO_3_]:[DFe] ratio ranged from <1 μM/nM at stations At7, At27, SCS, N18 and N7 to >30 μM/nM at stations EEP1 and EEP2 (Table 1 and Supplementary Tables S1, S5).

### Design of PCR and qPCR Primers

The previously published primer pairs *isiA*fw (WWAGNAR)/*isiA*rev (PYFADT) (with a product length of ∼860 bp) and *isiA*fw (WWAGNAR)/*isiA*rev (HLWHA) (with a product length of ∼1,000 bp), which were designed based on freshwater *isiA* genes (Geiss et al., 2001a,b), were first tested with our samples but both had poor amplification results. Therefore, we screened a total of 28 marine *Synechococcus* genomes for the *isiA* gene and designed new PCR primers. Out of those 28 genomes, 12 genomes harbored the *isiA* gene (Supplementary Table S3) and their translated IsiA protein sequences were aligned and screened manually for conserved regions. A completely conserved peptide sequence near the N-terminal (YDWWAG), modified from Geiss et al. (2001a,b), was chosen to design the degenerated forward primer *isiA*33F (Supplementary Figure S1). Several other conserved regions (213–218 bp, EDVMGG; 306–311 bp, PYFSDT; 346–351 bp, HFWHAL) were also found downstream the protein sequences (Supplementary Figure S1). The nucleotide sequences encoding these peptides were aligned, and reverse primers (e.g., *isiA*654R, *isiA*933R and *isiA*1053R) were designed after sequence comparisons. Testing on sample SIO-50m, the primer pair *isiA*33F (5′-ACY TAT GAC TGG TGG GC-3′) and *isiA*654R (5′-CCD CCC ATV ACR TCY TC-3′) generated the best amplification results in terms of diversity (Supplementary Figure S3) and thus was chosen for this study.

**FIGURE 2:**
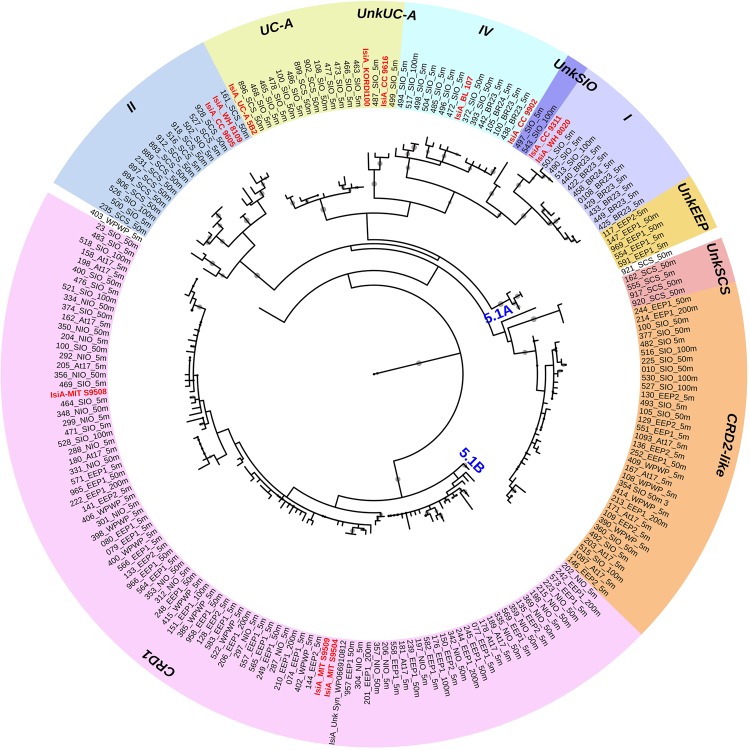
Circular maximum likelihood phylogenetic tree drawn based on 3% OTU (operational taxonomic units) cutoff of IsiA protein sequences. Each of the retrieved OTU is written in black in the form of clone number_station_depth, and known reference strains are indicated in red. Individual bootstrap values over 50% are indicated by circles of varying size on each branch. Gene phylotypes belonging to different *Synechococcus* clades are indicated by different colors. Further information on reference strains and the classification of each sequence can be found in Supplementary Tables S3, S4, respectively.

To assess the specificity of qPCR primers, an extra 28 homologous prochlorophyte-chlorophyll-binding *pcb* genes of 10 *Prochlorococcus* str. were recruited for screening together with the 12 *isiA* sequences (Supplementary Table S3 and Supplementary Figures S1, S2). A conserved region specific for *isiA* was manually found between 111 and 116 bp encoding the peptide sequence IWHTLR (Supplementary Figure S1), corresponding to their reverse gene primer *isiA*348R. This peptide sequence distinguishes IsiA from Pcb and the resulting qPCR product is short enough (∼300 bp) to yield high quantification efficiency. Applying the PCR and qPCR primers, ∼600 bp partial *isiA*/*pcb* fragments were amplified from all *isiA*-containing *Synechococcus* and some *Prochlorococcus* genomes, and ∼300 bp short *isiA* fragments were only retrieved from *isiA*-containing *Synechococcus* genomes, respectively (Supplementary Figure S2). Further sequencing of those fragments retrieved *isiA* genes covering all known *isiA*-harboring *Synechococcus* clades as well as unknown phylotypes (Figure 2 and Supplementary Table S4).

### Diversity and Distribution of *isiA* Genes

**FIGURE 3:**
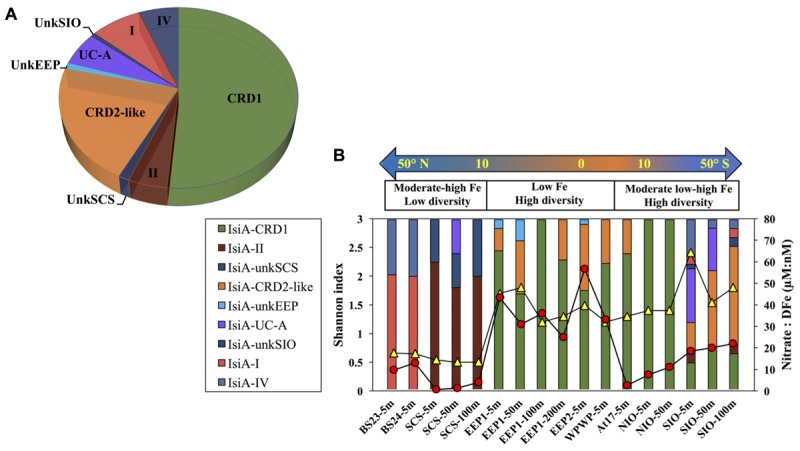
**(A)** Overall relative abundance of the nine *isiA* clades, across all stations. **(B)** Composition of *isiA* clades at each station arranged along a latitudinal gradient from 50° N to 50° S. The [NO_3_]:[DFe] ratio (red circles) and Shannon index of sequence diversity (yellow triangles) are also shown for each station.

A total of 440 *Synechococcus isiA* sequences were amplified from all stations except for 100 m and 200 m of NIO and 200 m of SCS and SIO (probably due to low *Synechococcus* numbers) and were assigned to nine phylotypes belonging to different *Synechococcus* clades (Figure 2, 3A). Using the previously identified *isiA* genes (Supplementary Table S3) and classification of *Synechococcus* clades (Saito et al., 2005; Scanlan et al., 2009; Mazard et al., 2012; Ahlgren et al., 2014), the most abundant *isiA* sequences in our study were from clade CRD1 (accounting for ∼50% of the total sequences; Figure 3A), a clade that was originally recruited in the upwelling Costa Rica dome and later widely discovered in upwelling tropical/subtropical oceans (Saito et al., 2005; Sohm et al., 2015; Farrant et al., 2016), with three closest known reference strains MIT S9504, MIT S9508, MIT S9509 (Supplementary Table S3) (Cubillos-Ruiz et al., 2017). The second-most abundant clade was a group without known references (yet-to-be cultivated) but frequently co-existed with clade CRD1, and thus likely clade CRD2 according to Ahlgren et al. (2014) and Sohm et al. (2015) or EnvB according to Farrant et al. (2016) (labeled as IsiA-CRD2-like in our study) (Figure 2, 3). Other commonly amplified *Synechococcus* that harbor *isiA* belonged to clades I, IV (commonly found in cold waters with higher nutrients) (Sohm et al., 2015) and UC-A (inhabiting oligotrophic ocean with relatively low abundance of the population) (Choi et al., 2014). The remaining *isiA* sequences comprised another known clade II (commonly found in warm and oligotrophic waters) (Sohm et al., 2015) and three small unknown clades of unkSCS (exclusively retrieved from SCS), unkSIO (exclusively retrieved from SIO), and unkEEP (exclusively retrieved from EEP).

**FIGURE 4:**
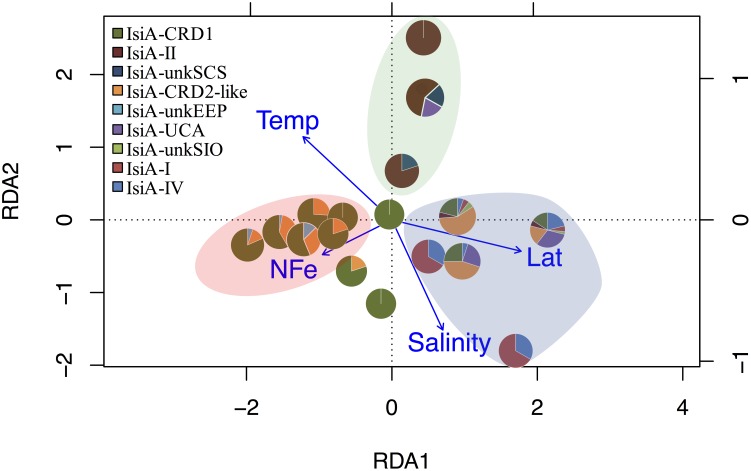
RDA tri-plot indicating the correlation between the *isiA* clades identified in this study and measured environmental parameters. Blue arrows denote the environmental variables that significantly correlated with sample ordination, and pie charts depict the relative abundances of the *isiA* clades at each station. Background shading in different colors points at three biogeographical clusters. The pink-beige cluster includes the low-Fe stations of EEP1, EEP2, and WPWP; the blue cluster includes the high-latitude cold stations of BS23, BS24, and SIO; the green cluster consists exclusively of the high-Fe and warm water at the three depths of station SCS.

Distinct variation in the diversity and composition of *isiA* genes was demonstrated along a latitudinal gradient, and among different iron habitats (as characterized by the [NO_3_]:[DFe] ratio) (Figure 3B). CRD1 and CRD2-like clades were most commonly retrieved together and dominant in 10°N–10°S warm waters, with high diversity of *isiA* genes (as represented by the Shannon diversity index). Although *isiA* genes of clades CRD1 and CRD2-like were obtained from a wide range of iron conditions, their distribution was mostly associated with high [NO_3_]:[DFe] ratios, as demonstrated by RDA (Figure 4). Clade II dominated at all three depths of station SCS, and all three depths were characterized by a low *isiA* diversity, and low [NO_3_]:[DFe] ratio (Figure 3B). The *isiA* genes of the high-latitude stations BS23 and BS24 with moderate-low [NO_3_]:[DFe] were co-dominated by clade I and clade IV, showed a low *isiA* diversity and in line with expectation that their *isiA* gene compositions at these stations was associated with low temperature and high latitude (Figure 3B, 4). By contrast, the three depths sampled at station SIO of moderate-high [NO_3_]:[DFe] ratios have also showed high *isiA* diversity, including all *isiA*-harboring clades (CRD1, CRD2, I, II, IV) recovered from other stations and also some clades unique to station SIO like UC-A and unkSIO. Yet, their *isiA* composition was also associated with low temperature and high latitude (Figure 3B, 4).

### *IsiA* Abundance and Iron Availability

In the surface waters (5 m depth) across all stations, *isiA* gene abundances were well above the detection limit of our qPCR assay at all stations, and ranged from 5.55 × 10^5^ gene copies L^-1^ at station At7 in the north tropical Atlantic Ocean to 2.72 × 10^7^ gene copies L^-1^ at station EEP2 in the eastern equatorial Pacific Ocean. At greater depths of 200 m at stations SCS, *isiA* gene abundance declined possibly below the detection limit of ∼800 gene copies L^-1^ (Supplementary Table S6). *Synechococcus* abundance ranged over ca. one order of magnitude in the surface, from 2.33 × 10^6^ cells L^-1^ at station SCS to 3.81 × 10^7^ cells L^-1^ at station NIO (Supplementary Table S6).

**FIGURE 5:**
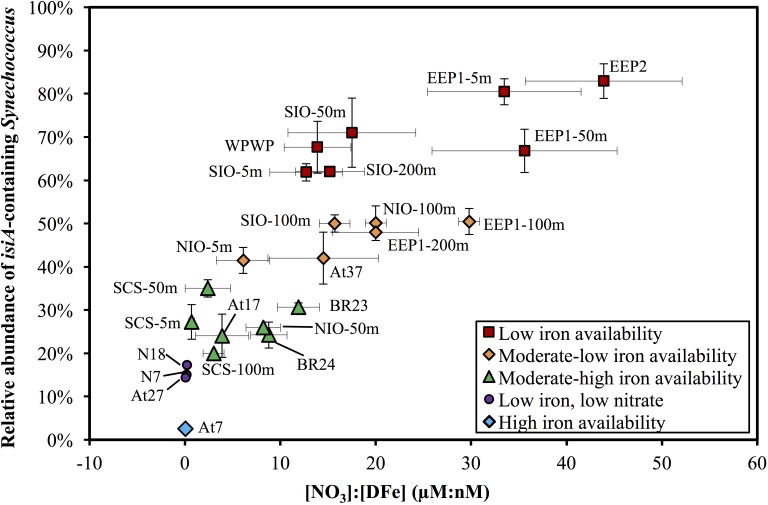
Scatter plot of the [NO_3_]:[DFe] ratio and the relative abundance of *isiA*-containing *Synechococcus* across the surface waters of all 14 stations and depth profile of four stations. Colored symbols represent five iron availability categories, see Supplementary Table S5 for the criteria used to distinguish these categories. Vertical and horizontal error bars on each dot represents the standard deviations of *isiA*:cell and [NO_3_]:[DFe], respectively.

The relative abundance of *isiA*-containing *Synechococcus, isiA*:cell, varied from <10% to >80% (Figure 5 and Supplementary Tables S5, S6). High *isiA*:cell (>50%) were observed in potentially iron-limited waters (with high [NO_3_]:[DFe] ratio) of the eastern equatorial Pacific (5 m and 50 m at station EEP1, 5 m at EEP2), western equatorial Pacific (5 m at station WPWP) and the southern Indian Ocean (5 m, 50 m, and 200 m at station SIO). Deeper water depths at station EEP1 (100 m and 200 m), SIO (100 m) and NIO (100 m), 5 m at station NIO in the Northern Indian Ocean and 5 m at station At37 in the southern Atlantic Ocean also revealed relatively high values (40–50%) of *isiA*:cell, followed by moderate to low values (20–40%) for the Bering Sea (stations BR23, BR24), SCS (5–100 m at station SCS), 50 m at station NIO and equatorial Atlantic Ocean (station At17). Lowest *isiA*:cell values (<10%) were observed in the iron rich water (with low [NO_3_]:[DFe] ratio) of the North Atlantic subtropical gyre (station At7). Interestingly, water sampled from environments with both low-Fe and low-N concentrations in the western part of the North Pacific subtropical gyre (stations N7 and N18) and the South Atlantic subtropical gyre (station At27) also showed low *isiA*:cell (Figure 5 and Supplementary Tables S5, S6).

Multiple regression analysis revealed a significant negative effect of iron (in nM) and a significant positive effect of nitrate (in μM) on the relative abundance of *isiA*-containing *Synechococcus* cells, according to the following regression model (*R*^2^ = 0.81, *n* = 24, *p* < 0.001):

$$\mathrm{Log}(\mathrm{isiA:cell})\;=\;-0.73\;\mathrm{[DFe]}\;+\;0.05\;[{\mathrm{NO}}_{3}]\;-\;0.33$$

The normal Q–Q plot of the standardized residuals indicates that the regression model fitted well to the data (Supplementary Figure S4). Interestingly, regressions with only iron (Log(*isiA*:cell) = -0.70[DFe] - 0.11) or only nitrate (Log(*isiA*:cell) = 0.05[NO_3_] - 0.68) were also significant (*P* < 0.001 and *P* < 0.01, respectively), but had a much lower explanatory power (*R*^2^ = 0.46 and *R*^2^ = 0.30, respectively; Supplementary Figures S5a,b). Hence, it is the combination of both iron and nitrate that determines the relative *isiA*-abundance. Other environmental variables (temperature, salinity, Chl *a*) were also fitted into the regression model, but none of these variables had a significant effect (*P* > 0.05; Supplementary Figures S5c–e). Altogether, variation in [DFe] and [NO_3_] explained more than 80% of the variation in the relative abundance of *isiA*-containing cells across stations and water depths.

## Discussion

### Biogeography of *isiA*-Containing Clades and “Low-Iron Specialists”

We characterized distinct sets of *isiA*-harboring *Synechococcus* that have adapted to different ocean habitats defined by iron availability (Figure 3B, 4). The most ubiquitous *isiA*-containing clades, CRD1 and CRD2-like, were not only recovered and dominant in low-Fe habitats of the eastern equatorial Pacific (EEP1 and EEP2), but also at stations with higher Fe availability in both the Indian Ocean (e.g., NIO) and Atlantic Ocean (e.g., At17). This is interesting as previous studies have suggested that clades CRD1 and CRD2 were restricted to low-Fe environments (Sohm et al., 2015). Globally dominant *Synechococcus* clades, such as clades II (and possible III), were retrieved from some stations but not as commonly as CRD1 and CRD2-like. We therefore assume clades II and III have selectively lost or not gained the *isiA* gene during evolution. Indeed, all known genomes of clade III (e.g., WH8102, WH8103) and several clade II strains (e.g., KORDI-52) lack the *isiA* gene (Rivers et al., 2009; Scanlan et al., 2009) (Supplementary Table S3). This is in line with previous studies that have found no evidence for “low-Fe specialists” in those clades (Sohm et al., 2015; Farrant et al., 2016). One could assume that those species of clade II and III are likely to benefit from episodic iron enrichments whereas clade CRD1 and CRD-2like tend to proliferate in low-iron environments.

In addition to the more ubiquitous *isiA*-containing clades, several other *isiA* phylotypes were exclusively recovered from specific stations, such as unkEEP from the low-iron eastern equatorial Pacific (stations EEP1, EEP2), and thus seemed to have specifically adapted to that *in situ* ecological niche. At high-latitude stations (e.g., the Bering Sea), the known cold-water prevalent clades I and IV were the dominant *isiA*-containing *Synechococcus*. These results are generally in agreement with previous studies on cyanobacterial biogeographical distribution (Zwirglmaier et al., 2008; Huang et al., 2011; Mazard et al., 2012; Ahlgren et al., 2014; Sohm et al., 2015), especially the recently proposed close relationship between finer (than clade level) “ecologically significant taxonomic units” (ESTU) and specific ecological niches driven by temperature, iron, phosphate (Farrant et al., 2016) and light (Grébert et al., 2018) through the Tara ocean expedition. Interestingly, the high diversity of *isiA*-harboring *Synechococcus* revealed from station SIO in the present study, is consistent with the various low-iron “specialist” cyanobacteria observed earlier at an adjacent station east of Madagascar (TARA_052) (Farrant et al., 2016). This may suggest a special habitat off Madagascar supporting the prevalence of *isiA*-harboring and other genetically tuned low-iron adapted cyanobacteria.

### Relative Abundance of *isiA* Gene Varies With Iron and Nitrate

Through our *isiA* gene quantification study, we have demonstrated a strong relationship between the relative abundance of *isiA*-containing *Synechococcus* cells and the iron and nitrate concentrations across a diverse range of ocean environments in the Atlantic, Pacific, and Indian Oceans. Specifically, the relative abundance of the *isiA* gene is negatively related with iron availability but positively related with nitrate availability. For instance, HNLC regions (e.g., stations EEP1 and EEP2) in which macronutrients such as nitrate are in ample supply but iron is limiting have a high relative abundance of the *isiA* gene. These results indicate that the presence of the *isiA* gene provides a clear selective advantage for *Synechococcus* assemblages living in iron-limited but nitrate-replete environments. In comparison, the *isiA* gene does not appear to provide a selective advantage in high-iron habitats (e.g., in the North Atlantic subtropical gyre), where the relative abundance of *isiA*-containing *Synechococcus* cells is low. Likewise, environments are limited by both nitrate and iron, e.g., stations N7 and N18 (Li et al., 2015), have a low relative abundance of the *isiA* gene as well, suggesting that the *isiA* gene does not offer a selective advantage in environments where nitrogen is limiting. These results indicate that both iron and nitrate exert a major selection pressure on the presence of the *isiA* gene in cyanobacterial populations.

Our results are consistent with previous studies pointing out that macronutrients such as nitrate must be considered when using *isiA* and other iron-responsive genes as biomarkers of iron limitation (Behrenfeld et al., 2006; Schrader et al., 2011; Mackey et al., 2015). Laboratory experiments have shown that accumulation of the IsiA protein is substantially less when experiencing synergetic limitation of nitrogen and iron rather than iron limitation alone (Schrader et al., 2011), and physiological signatures of iron limitation were only observed when nitrate is replete (Moore et al., 2013; Browning et al., 2017). The high relative abundance of the *isiA* gene that we found in HNLC regions is in agreement with the distinct fluorescence signatures reported from these HNLC regions, which have commonly been attributed to the accumulation of chlorophyll-binding proteins such as IsiA (Behrenfeld et al., 2006, 2009; Schrader et al., 2011; Behrenfeld and Milligan, 2013; Macey et al., 2014). Our results therefore strengthen the argument proposed by Behrenfeld et al. (2006, 2009) and Behrenfeld and Milligan (2013), that it is important to separate the functionally uncoupled chlorophyll molecules bound by IsiA from functional chlorophyll molecules for accurate primary production estimates, especially in HNLC areas.

Some laboratory studies indicated an increased expression of the *isiA*(BC) operon at high salt stress (Vinnemeier et al., 1998), heat stress (Vinnemeier et al., 1998; Kojima et al., 2006) and high light stress (Havaux et al., 2005). However, these studies were performed with freshwater *Synechocystis* and thus may not be relevant to marine *Synechococcus* species. Furthermore, these laboratory studies applied extremely high salinities, irradiance and temperatures that are not encountered in the oceans. In our field data, we found a significant relation between *isiA* and iron and nitrate availability, but the relative abundance of *isiA* did not show a significant relationship with salinity or temperature (Supplementary Figures S5c–e). Furthermore, the relative abundance of *isiA* was not positively associated with high light conditions either (most likely happens in the very surface water), as the relative abundance of *isiA*-containing *Synechococcus* cells tend to remain rather constant or even slightly increased with depth at several stations vertically sampled (SCS, SIO, NIO, EEP1; Supplementary Figure S6).

Our results suggest that the relative abundance of the *isiA* gene can be added to the ‘toolbox’ of molecular markers assessing the iron nutritional status of marine phytoplankton. Similar molecular approaches have previously been applied to the diatom *Thalassiosira oceanica* (Chappell et al., 2015) and marine cyanobacteria (Webb et al., 2001; Chappell et al., 2012), based on expression differences of known genes involved in acclimation to iron stress. Such biological responses at the gene level can be a powerful proxy for iron bioavailability, which is sometimes difficult to assess by chemical measurements alone because of the complex speciation of iron and its interactions with other molecules (Chappell et al., 2015; Browning et al., 2017).

## Conclusion

In conclusion, this study provides novel information on the biogeographical distribution of the photophysiologically important *isiA* gene. Consistent with earlier results of a more limited geographical extent (e.g., Bibby et al., 2009), we found a close relationship with iron bioavailability in various ocean environments. More specifically, of all investigated oceanographic parameters, only iron and nitrate availability were strongly correlated with the relative abundance of *isiA*.

Therefore, we propose here that the relative abundance of *isiA*-containing *Synechococcus* cells (*isiA*:cell) is driven by adaptation to iron limitation and thus can be used as a potential biomarker for iron limitation under nitrate replete conditions. Obtaining estimates of the relative abundance of *isiA*-containing cells through qPCR could aid future iron limitation studies given the challenges of frequent contamination in trace-metal-clean techniques (Fitzwater et al., 1982) and the extremely complex speciation of iron ligands (Hutchins et al., 1999). Further laboratory work investigating iron-related physiology of IsiA proteins using model strains of the CRD1 and CRD2 clades will also provide exciting information on these special photosynthetic strategies, and how they enable these clades to dominate in low-iron environments. Lastly, an understanding of the environmental controls of the biogeography of *isiA* is useful for interpretation of the biophysical signatures of iron limitation that can be collected remotely and used to map the extent and dynamics of iron limitation in the ocean.

## Author Contributions

QL and NJ designed the study and developed the experimental methods. QL conducted the experiments and drafted the manuscript. JH and TB helped with interpretation of the results and manuscript revision.

## Conflict of Interest Statement

The authors declare that the research was conducted in the absence of any commercial or financial relationships that could be construed as a potential conflict of interest.

## Footnotes

1 https://giovanni.gsfc.nasa.gov/giovanni/

2 https://engaging-web.mit.edu/~jahn/pub/ecco2darwin_iron_monthly.nc

3 https://raxml-ng.vital-it.ch/

4 http://itol.embl.de/
