# Supplementary material for: Biogeography of Cyanobacterial isiA Genes and Their Link to Iron Availability in the Ocean
Source: Front Microbiol. 2019 Apr 4;10:650. doi: 10.3389/fmicb.2019.00650 (PMC6460047; doi:10.3389/fmicb.2019.00650)

Supplementary Fig. S1. Alignments of IsiA and Pcb protein sequences. The black and red arrows indicate the binding sites of the potential and chosen primers to amplify isiA genes, respectively.

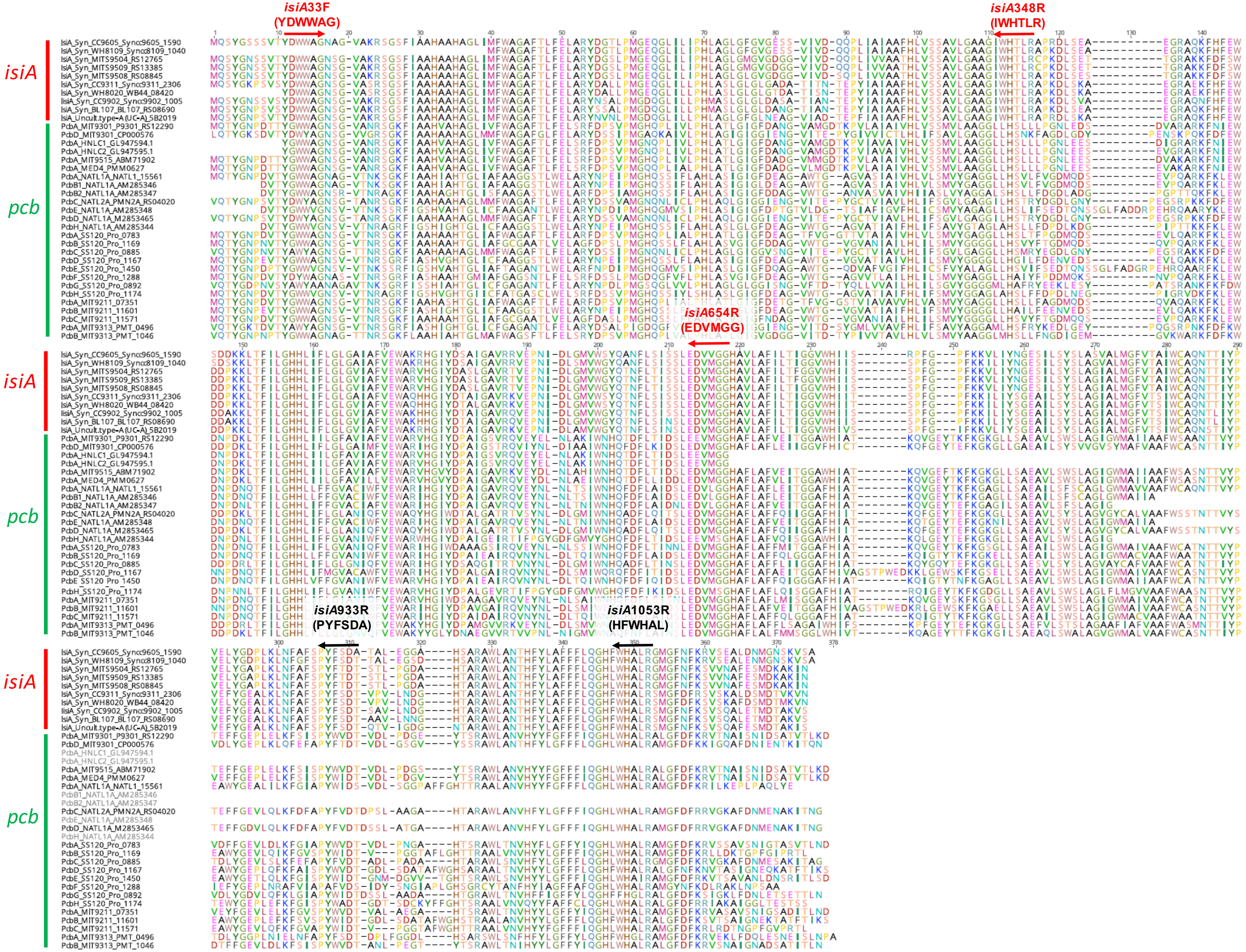

Supplementary Fig. S2. Results of mapping our PCR primer pair isiA33F & isiA654R and qPCR primer pair isiA33F & isiA348R to a collection of marine Synechococcus and Prochlorococcus genomes (Supplementary S3). The red and green arrows point out the forward and reverse primer binding sites on the genome, respectively. Those Synechococcus genomes with no primer bindings suggest the absent of *isiA* gene, and none of the Prochlorococcus genome generated binding products of the isiA specific qPCR primers (isiA33F & isiA348R).

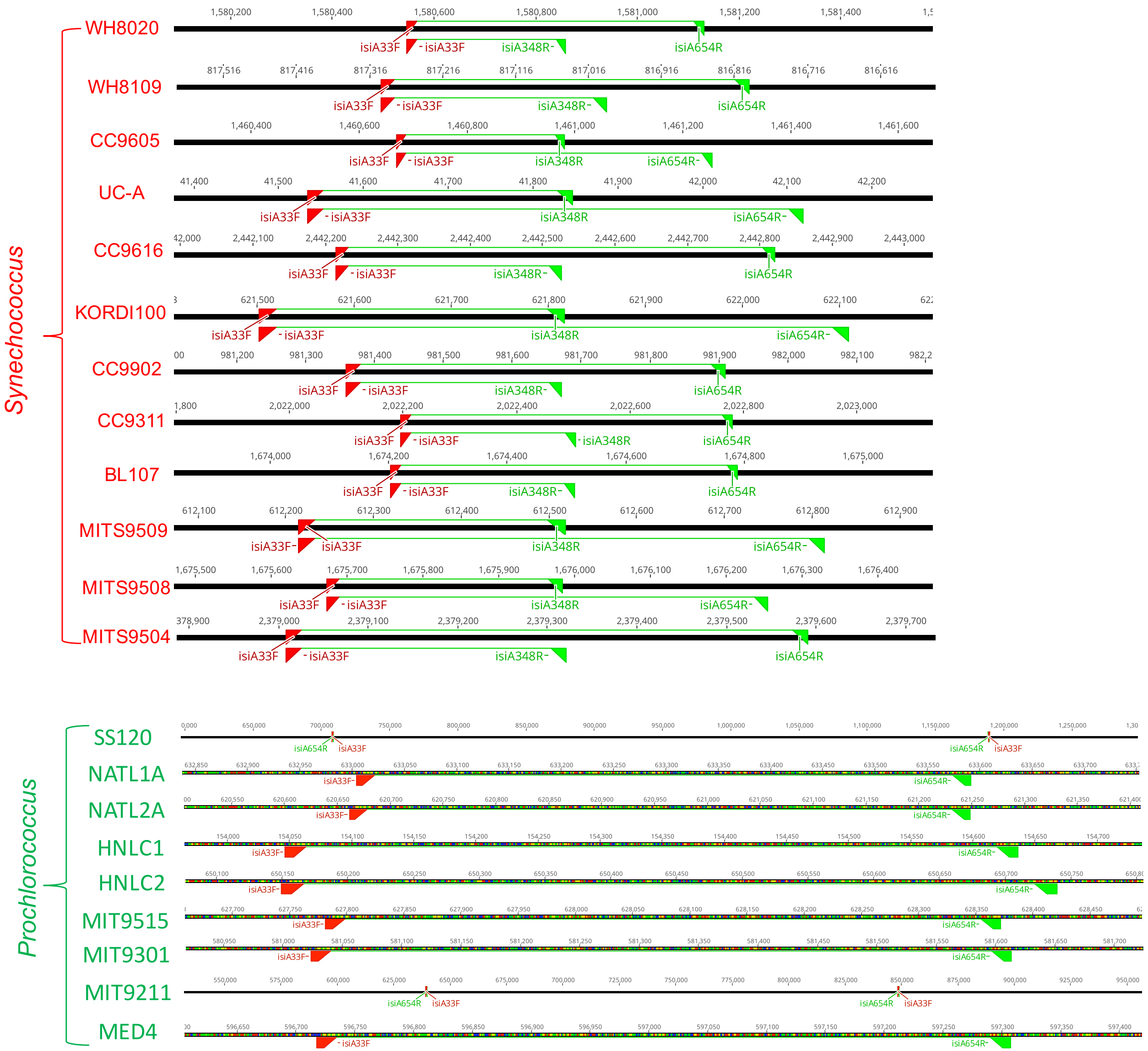

Supplementary Fig. S3. Neighbor-joining phylogenetic tree of *isiA* gene sequences retrieved from 50 m depth of station SIO, using 3 different primer pairs designed for marine *Synechococcus isiA* genes (see Results). The primer pair (*isiA*33F/*isiA*654R) further used in this study recovered the highest *isiA* diversity among all investigated primer pairs.

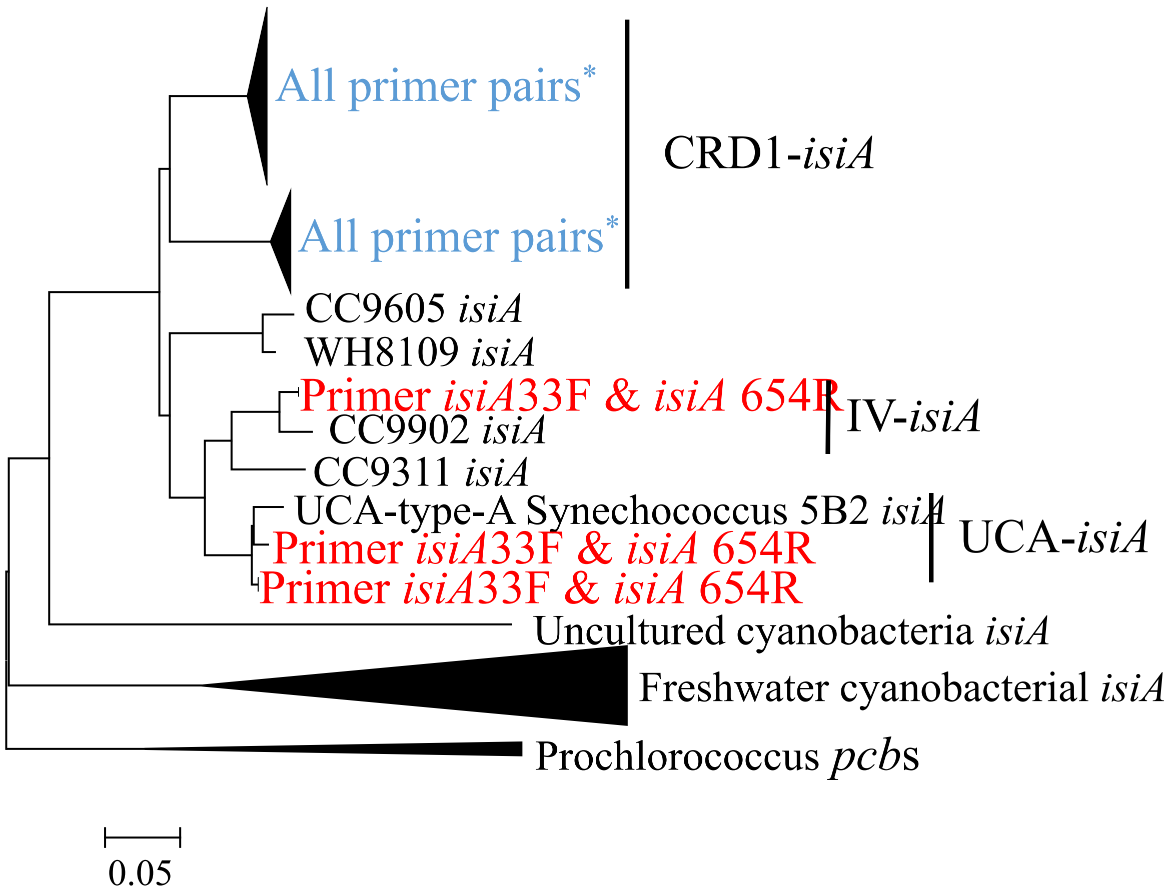

* isiA33F and reverse primer pairs *isiA*654R, *isiA*933R and *isiA*1053R

Supplementary Fig. S4. Normal Q-Q plot of the standardized residuals of the regression model Log(isiA:cell) = − 0.73 [DFe] + 0.05 [NO3] − 0.33.

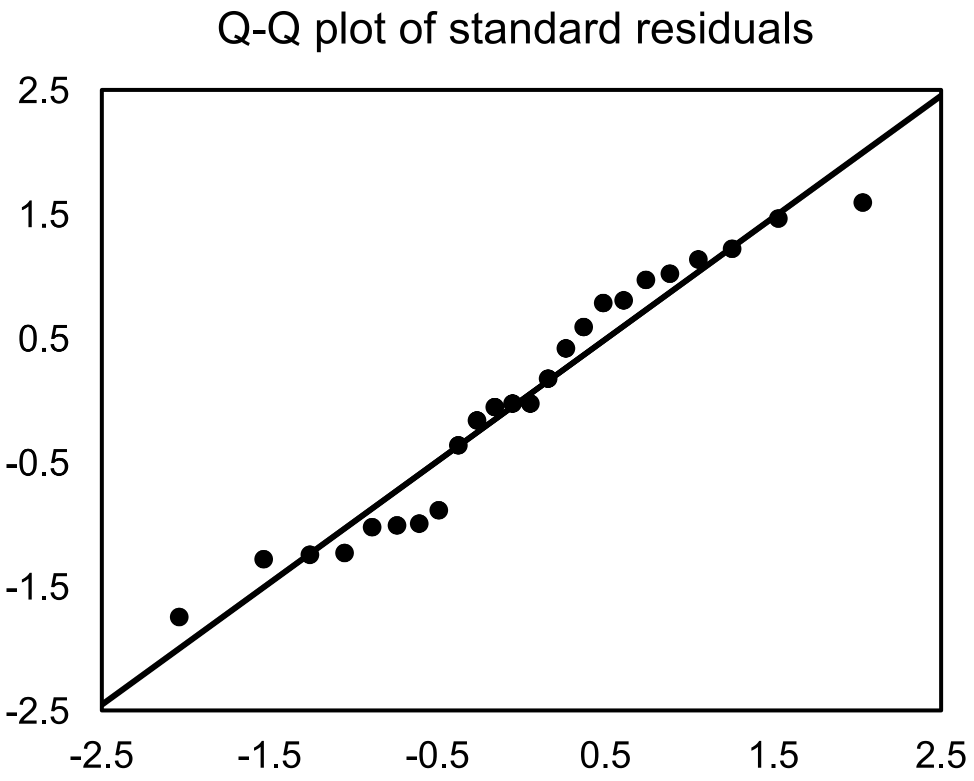

Supplementary Fig. S5. Relationships between the relative abundance of *isiA* (expressed as Log(*isiA*:cell)) and (a) [DFe], (b) [NO_3], (c) temperature, (d) chlorophyll *a* and (e) salinity across all 14 stations (n=24).

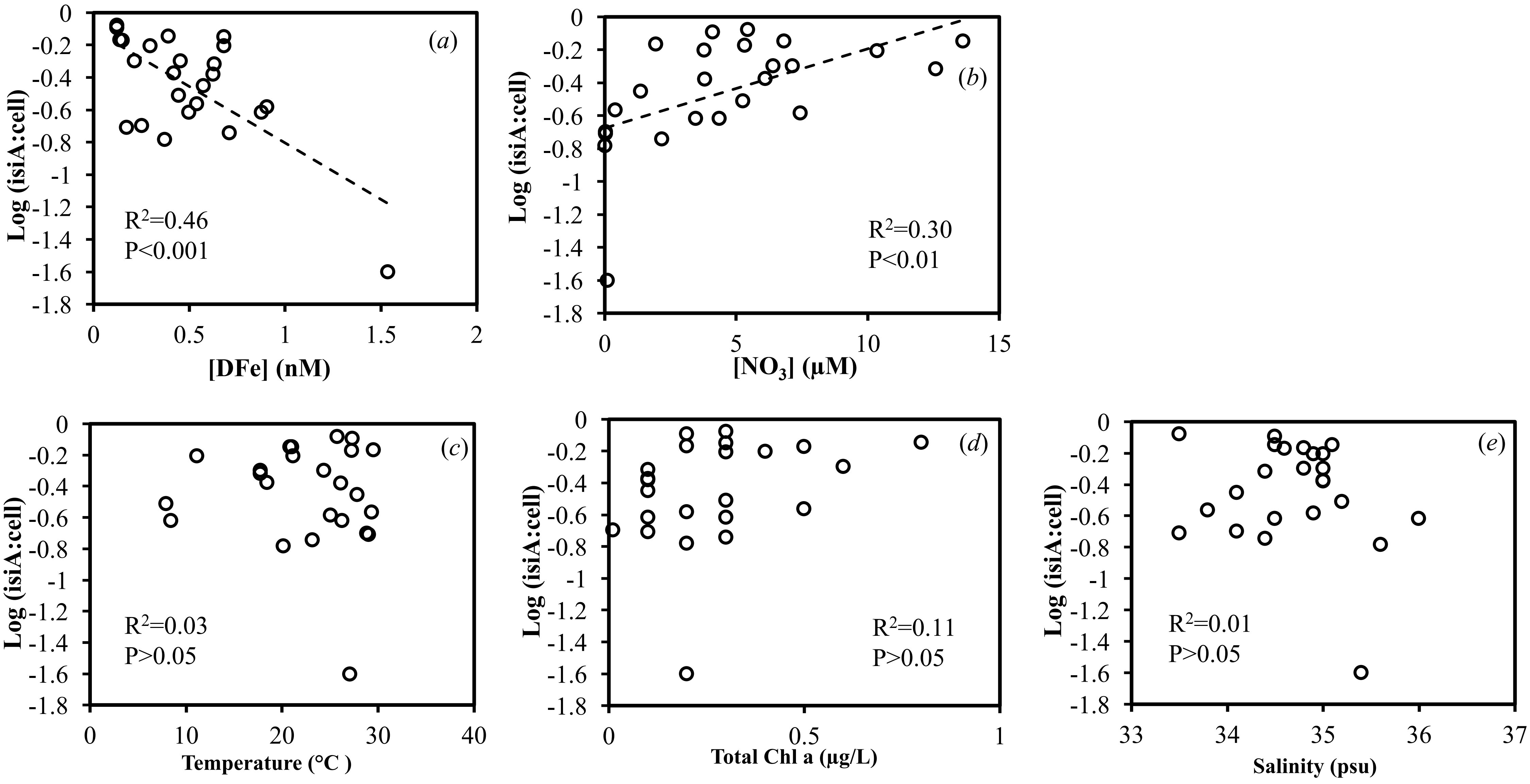

Supplementary Fig. S6. Vertical distribution of the *isiA*:cell ratio, *Synechococcus* abundance and *isiA* gene abundance at stations EEP1, SCS, SIO and NIO. Red symbols denote individual measurements at the different stations and black symbols represent the average values over all 4 stations.

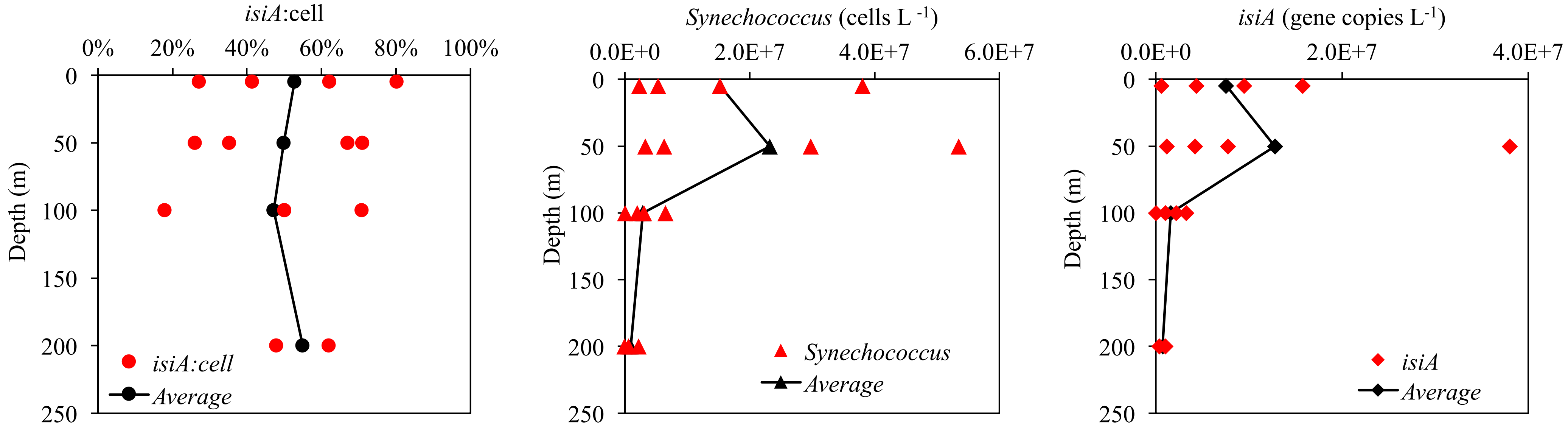

Supplement: Supplementary file 2 [file Data_Sheet_2.pdf]
